# DNA Damage in Rheumatoid Arthritis: An Age-Dependent Increase in the Lipid Peroxidation-Derived DNA Adduct, Heptanone-Etheno-2′-Deoxycytidine

**DOI:** 10.1155/2013/183487

**Published:** 2013-10-07

**Authors:** Masako Ogawa, Tomonari Matsuda, Atsushi Ogata, Toshimitsu Hamasaki, Atsushi Kumanogoh, Toshihiko Toyofuku, Toshio Tanaka

**Affiliations:** ^1^Department of Respiratory Medicine, Allergy and Rheumatic Diseases, Osaka University Graduate School of Medicine, 2-2 Yamada-Oka, Suita, Osaka 565-0871, Japan; ^2^Research Center for Environmental Quality Management, Kyoto University, 1-2 Yumihama, Otsu, Shiga 520-0811, Japan; ^3^Department of Immunopathology, WPI Immunology Frontier Research Center, Osaka University, 3-1 Yamada-Oka, Suita, Osaka 565-0871, Japan; ^4^Department of Biomedical Statistics, Osaka University Graduate School of Medicine, 2-2 Yamada-Oka, Suita, Osaka 565-0871, Japan; ^5^Department of Clinical Application of Biologics, Osaka University Graduate School of Medicine, 2-2 Yamada-Oka, Suita, Osaka 565-0871, Japan

## Abstract

*Objective*. To evaluate what types of DNA damages are detected in rheumatoid arthritis (RA). *Methods*. The DNA adducts such as 8-oxo-hydroxy-7,8-dihydro-2′-deoxyguanosine (8-oxo-dG), 1,N^6^-etheno-2′-deoxyadenosine (**ε**dA), and heptanone-etheno-2′-deoxycytidine (H**ε**dC) in genomic DNAs, derived from whole blood cells from 46 RA patients and 31 healthy controls, were analyzed by high-performance liquid chromatography tandem mass spectrometry, and their levels in RA patients and controls were compared. In addition, correlation between DNA adducts and clinical parameters of RA was analyzed. *Results*. Compared with controls, the levels of H**ε**dC in RA were significantly higher (*P* < 0.0001) and age dependent (*r* = 0.43, *P* < 0.01), while there was no significant difference in 8-oxo-dG and **ε**dA accumulation between RA patients and controls. H**ε**dC levels correlated well with the number of swollen joints (*r* = 0.57, *P* < 0.0001) and weakly with the number of tender joints (*r* = 0.26, *P* = 0.08) of RA patients, while they did not show a significant association with serological markers such as C-reactive protein and matrix metalloproteinase 3. *Conclusion*. These findings indicate that H**ε**dC may have some influence on the development of RA and/or its complications.

## 1. Introduction

Rheumatoid arthritis (RA) is a systemic, chronic inflammatory disease of the joints and surrounding tissues, accompanied by intense pain, irreversible joint destruction, and systemic complications [1]. Its etiology has not been fully clarified yet, but oxidative stress is one of the pathological factors, which contribute to its development [2–5]. Shao et al. recently reported detecting the presence of DNA damage and deficiency of the DNA repair enzyme, ataxia telangiectasia mutated (ATM), in RA T cells [6]. Moreover, a DNA oxidation product, 8-oxo-hydroxy-7,8-dihydro-2′-deoxyguanosine (8-oxo-dG), has been shown to be highly expressed in RA patients [2]. DNA damage induced by exposure to ionizing radiation, ultraviolet light, and exogenous or endogenous chemical mutagens causes DNA strand breakages, which are thought to be mutagenic, carcinogenic, and aging factors [2]. Additionally as new DNA damage, lipid peroxidation-derived DNA adducts have been recently reported in human [7], and lipid peroxidation has been also considered as a factor of the pathogenesis or the local inflammatory response of RA [4, 8–10]. Representative DNA adducts are 8-oxo-dG, 1,N^6^-etheno-2′-deoxyadenosine (*ε*dA), and heptanone-etheno-2′-deoxycytidine (H*ε*dC); the latter two of which are direct reactive oxygen species- (ROS-) derived and lipid peroxidation-derived adducts. H*ε*dC is also deemed a 4-oxo-2(*E-*) nonenal (4-ONE-) generated product [7]. 

On the basis of these previous findings, in this study, in order to investigate the role of DNA adducts in the development of RA, we examined the amount of three DNA adducts, 8-oxo-dG, *ε*dA, and H*ε*dC, in whole blood cells from RA patients and healthy controls by means of a sensitive technique, high-performance liquid chromatography tandem mass spectrometry (LC-MS/MS). 

## 2. Materials and Methods

### 2.1. Study Participants

The study population consisted of 46 RA (38 women and 8 men) patients and 31 healthy controls (14 women and 17 men). All RA patients, age of median (range): 52 (22–81) years, met the 1987 revised American College of Rheumatology (ACR) classification criteria for RA [11]. The median (range) number of tender joints and swollen joints were 2 (0–28) and 2 (0–12), respectively, while the median (range) serum C-reactive protein (CRP) concentration was 0.83 (0.04–6.2) mg/dL (Table 1). Patient records showed that 19 had been treated with nonsteroidal anti-inflammatory drug (NSAID) (one with celecoxib, a selective cyclooxygenase 2 (Cox-2) inhibitor and 18 with other nonselective Cox-2 inhibitors), 29 with methotrexate, 29 with prednisolone, and 5 with biologics (two with etanercept and one each with infliximab, adalimumab, and tocilizumab). The control group comprised 31 healthy volunteers without any chronic disease, with the median (range) age of 36 (22–57) years. The subjects' written consent was obtained according to the Declaration of Helsinki, and the study has been approved by the ethics committee of Osaka University Hospital. The clinical findings and laboratory data from RA patients were obtained on the same day that the peripheral blood samples were drawn, so that some laboratory test data were missing.

### 2.2. DNA Purification and Digestion

Genomic DNA was purified from whole blood cells with the DNA Extractor WB Kit using the Sodium Iodide method (Wako, Osaka, Japan) with the addition of deferoxamine mesylate (Sigma Aldrich Japan KK, Tokyo, Japan) to all solutions for adjustment to a final concentration of 0.1 mM in accordance with the manufacturer's protocol, after which the purified DNA was stored at −80°C. Isolated DNA was digested to nucleosides with the nuclease P1 method previously described [12]. For DNA adduct analysis, 50 *μ*L of 30% dimethyl sulfoxide was added to each of the DNA samples and then subjected to LC-MS/MS.

### 2.3. DNA Adduct Standards and Stable Isotope Standards

**Figure 1:**
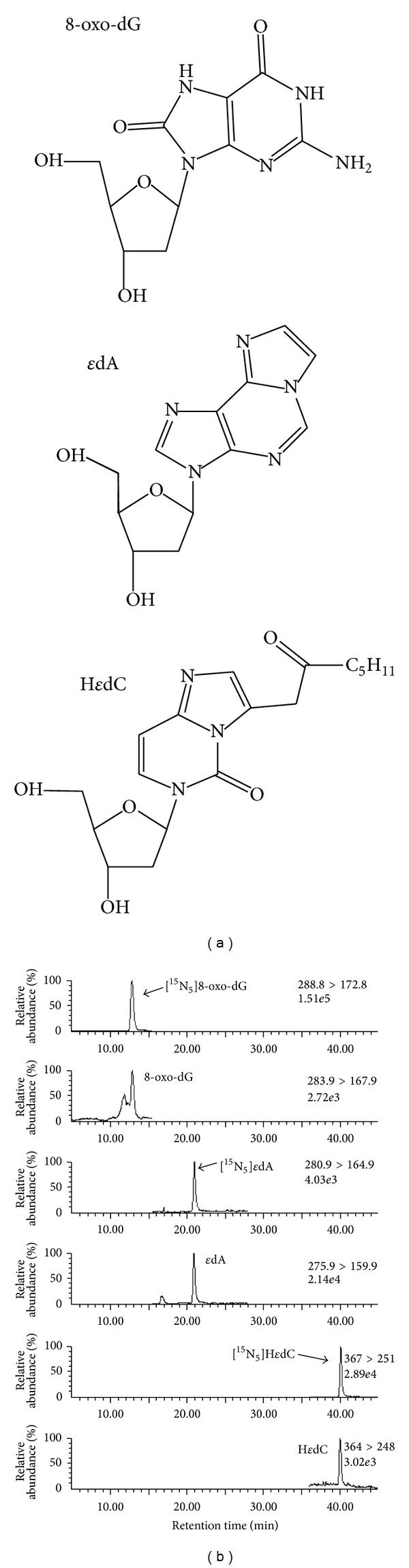
Chemical structures of DNA adducts and their corresponding peaks detected by LC-MS/MS. (a) Chemical structures of the DNA adducts 8-oxo-hydroxy-7,8-dihydro-2′-deoxyguanosine (8-oxo-dG), 1,N^6^-etheno-2′-deoxyadenosine (*ε*dA), and heptanone-etheno-2′-deoxycytidine (H*ε*dC). (b) DNA adducts were quantified by calculating the ratio of the peak of the DNA adduct to that of its standard isotope. The respective peaks of the standard isotope and the corresponding sample are shown.

Values for the three DNA adducts, 8-oxo-dG, *ε*dA, and H*ε*dC, were assessed and their chemical structures are shown in Figure 1(a). The 4-ONE DNA adduct H*ε*dC was synthesized according to the previously published methods [13]. 8-oxo-dG and *ε*dA were obtained from Sigma Aldrich Japan. [U-^15^N_5_]-8-oxo-dG was kindly provided by Dr. Shinya Shibutani, State University of New York, Stony Brook, NY, USA, and other DNA adduct stable isotope standards were synthesized according to the previously described methods [12] using [U-^15^N_5_]- or [U-^15^N_3_]-deoxynucleoside purchased from Cambridge Isotope Laboratories (Andover, MA, USA).

### 2.4. LC-MS/MS Instrumentation

Chromatography was performed with a Quattro Ultima Pt triple stage quadrupole mass spectrometer (Waters-Micromass, Milford, MA, USA) equipped with the alliance 2695 separation module, and a 2487 Dual *λ* Absorbance Detector (Waters, Milford, MA, USA) was used. An aliquot (20 *μ*L) of the digested DNA sample was injected and separated with the Shim-Pack XR-ODS column (3.0 mm × 75 mm, Shimadzu, Japan), eluted in a linear gradient of 5% to 30% methanol in water between 0 and 27 min, of 30% to 80% between 27 and 35 min, and then kept in 80% methanol between 35 and 40 min at a flow rate of 0.2 mL/min. The following experimental conditions were used: ion source temperature: 130°C, desolvation temperature: 380°C, cone voltage: 35 V, and collision energy: 15 eV, desolvation gas flow rate: 700 L/h, cone gas flow rate: 35 L/h, collision gas: argon. The positive ion mode was used for multireaction monitoring (MRM) analysis under the following conditions (cone voltage, collision energy, and precursor ion→product ion): [U-^15^N_5_]-8-oxo-dG: (40, 12, 288.8→172.8), [U-^15^N_5_]-*ε*dA: (35, 14, 280.9→164.9), [U-^15^N_3_]-H*ε*dC: (35, 10, 367.0→251.0), 8-oxo-dG: (40, 12, 283.9→167.9), *ε*dA: (35, 14, 275.9→159.9), and H*ε*dC: (35, 10, 364.0→248.0).

### 2.5. DNA Adduct Quantification

Each of the DNA adducts was quantified by calculating the ratio of the peak area of the target adducts to that of its isotope as follows: peak area of the potential DNA adduct/peak area of the internal standard/amount of 2′-deoxyguanosine (dG). The amount of dG in each DNA sample was estimated by monitoring the dG peak area at 254 nm with the UV-visible detector connected in series with LC-MS/MS system. QuanLynx (ver. 4.0) software (Waters-Micromass, Manchester, UK) was used to create standard curves and calculate the adduct concentrations. 

### 2.6. Statistics

Values for 8-oxo-dG, *ε*dA, and H*ε*dC in RA patients and healthy controls were compared by using the mean of analysis of covariance (ANCOVA), where the model included age and sex as covariates. Before this analysis, the values were log-transformed because 8-oxo-dG, *ε*dA, and H*ε*dC were not normally distributed. The results were back-transformed and then expressed as adjusted geometric mean ratios and their 95% confidence interval (CI). Associations between the levels of DNA adducts and clinical or laboratory test findings observed in RA patients were analyzed with the Spearman rank correlation coefficient. *P* values less than 0.05 were considered significant. All the analyses were performed with the SAS version 9.3 for Windows (SAS Institute, Cary, NC, USA). To match the number of sex between RA patients and controls, 14 women and 7 men were randomly chosen. For comparison of 8-oxo-dG, *ε*dA, and H*ε*dC in RA patients and healthy controls, the two-sample test was conducted for log-transformed data. In addition, the bootstrap method was used to generate the 10,000 sets of sample matched by sex, and each sample was analyzed by two-sample test to assess the robustness of the conclusion from the matched analysis. 

## 3. Results

### 3.1. Detection of 8-oxo-dG, *ε*dA, and H*ε*dC

Purified DNAs from peripheral whole blood cells from RA patients (*n* = 46) and controls (*n* = 31) were subjected to LC-MS/MS for the detection of specific DNA adducts. Several major peaks of DNA adducts were observed, and among these, the peaks corresponding to 8-oxo-dG, *ε*dA, and H*ε*dC and the stable isotope internal standards are shown in Figure 1(b). The volume of these DNA adducts was calculated with the method described in Section 2. 

### 3.2. H*ε*dC Is Elevated in Whole Blood Cells of RA Patients

**Figure 2:**
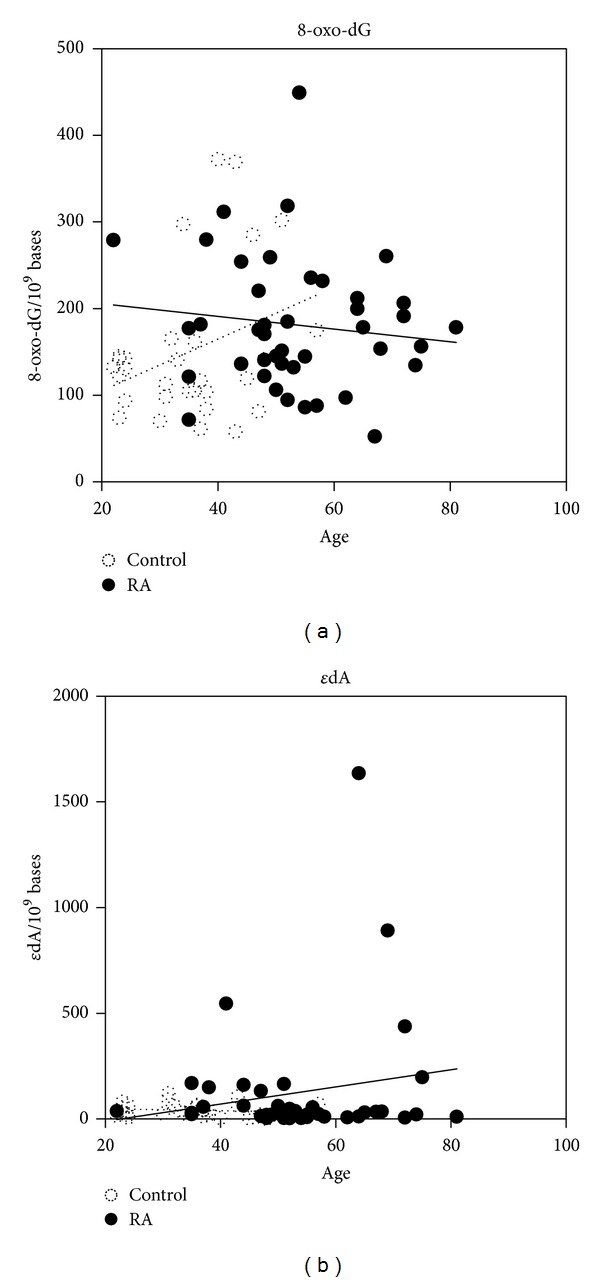
8-oxo-dG and *ε*dA levels in blood cells from RA patients and controls show no differences. (a) The respective median (range) values of 8-oxo-dG from RA patients and controls were 176.4 (52.5–449) and 127.1 (58.1–372) per 10^9^ bases. The straight and dotted lines represent linear approximation of the values of 8-oxo-dG versus age from RA patients and controls. (b) The respective median (range) values of *ε*dA from RA patients and controls were 29 (2.6–1635) and 34.7 (0.2–121) per 10^9^ bases. The straight and dotted lines represent linear approximation of the values of *ε*dA versus age from RA patients and controls.

**Figure 3:**
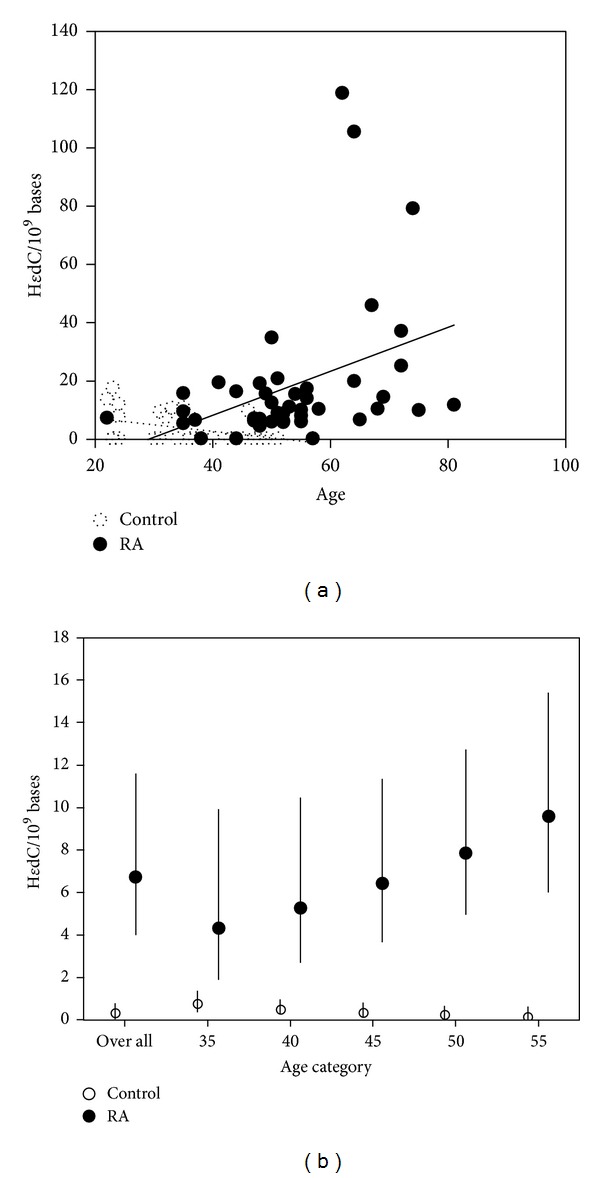
H*ε*dC levels are significantly elevated in RA patients. (a) The respective median (range) values of H*ε*dC from RA patients and controls were 10.3 (0.3–119) and 0.33 (0.3–17.8) per 10^9^ bases. H*ε*dC levels in RA patients were thus significantly higher than in controls (*P* < 0.0001). The straight and dotted line represent linear approximation of the values of H*ε*dC versus age from RA patients and controls. (b) The geographic mean level of H*ε*dC increased with aging in RA patients (*P* = 0.003).

The results for 8-oxo-dG and *ε*dA are shown in Figure 2. The median (range) levels of 8-oxo-dG per 10^9^ bases in RA patients and controls are 176.4 (52.5–449) and 127.1 (58.1–372), respectively, and they did not differ. Moreover, there was no significant difference in *ε*dA between the two groups, RA: 29 (2.6–1635) (median (range)) per 10^9^ bases versus control: 34.7 (0.2–121) per 10^9^ bases. Four patients with RA showed an increase in *ε*dA accumulation, but no association was detected between these values and disease activity parameters such as CRP and the number of involved joints. However, H*ε*dC levels were significantly higher in RA patients, 10.3 (0.3–119) (median (range)) per 10^9^ bases than those in controls, 0.33 (0.3–17.8) per 10^9^ bases (*P* < 0.0001) (Figure 3(a)), and this significance was observed in all age-interval analyses (mean ratio at 35: *P* = 0.0009; for other age intervals: *P* < 0.0001) as shown in Figure 3(b), and the mean of level of the adduct H*ε*dC in RA patients was approximately five times higher than that in controls. Moreover, in order to match the number of sex between RA patients and controls, the datum of 14 women and 7 men were randomly selected, and it was confirmed that H*ε*dC (but not 8-oxo-dG and *ε*dA) levels were significantly higher in RA patients than those in controls (*P* < 0.0001, data not shown).

### 3.3. Significant Positive Association between H*ε*dC Levels and Number of Swollen Joints and Aging

**Figure 4:**
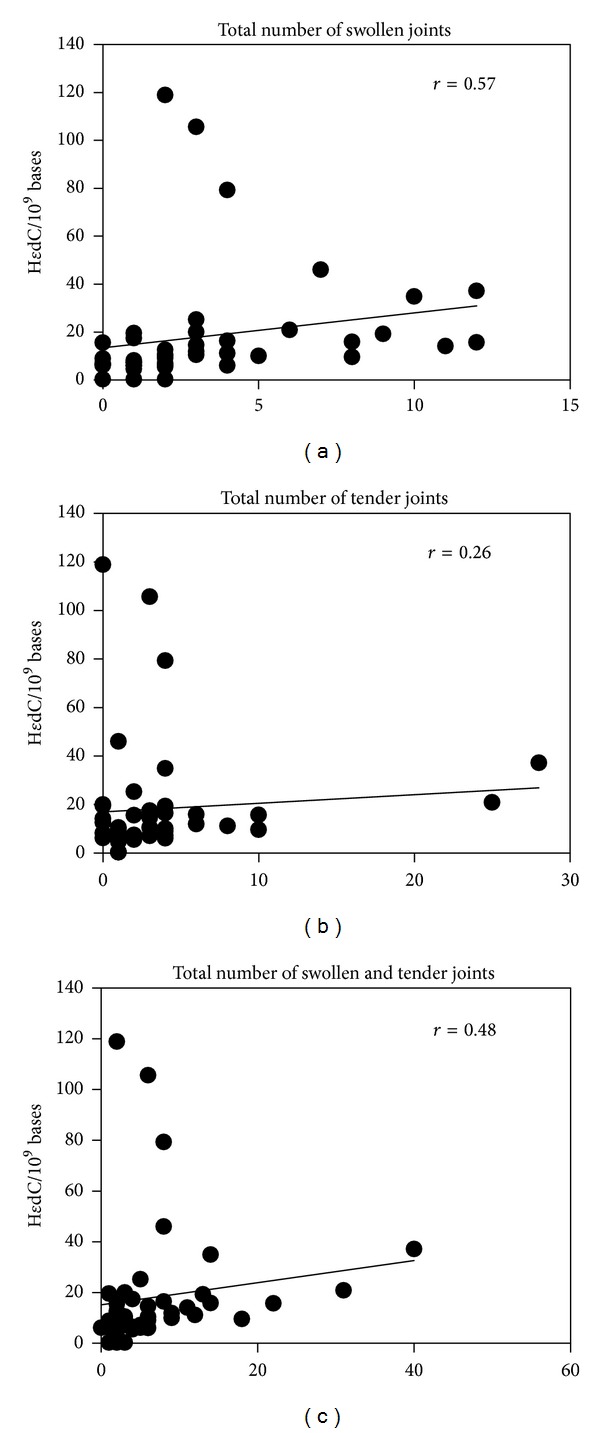
H*ε*dC levels show positive correlation with the number of swollen joints and the total number of swollen plus tender joints. H*ε*dC levels correlated weakly with the number of tender joints (*P* = 0.08) but strongly with that of swollen joints (*P* < 0.0001) and the total number of tender + swollen joints (*P* = 0.0005). The straight line represents linear approximation of the values of H*ε*dC versus the number of involved joints in RA patients.

Correlation analyses were performed to explore whether H*ε*dC levels correlated with clinical or laboratory test findings including platelet count, concentrations of CRP, serum amyloid A (SAA), albumin, and matrix metalloproteinase 3 (MMP3), as well as levels of total cholesterol, triglyceride, high-density lipoprotein cholesterol, and low-density lipoprotein cholesterol in addition to the number of swollen joints and tender joints. The results are shown in Table 2. Slightly positive, but not statistically significant, correlations were observed between the levels of H*ε*dC and the number of platelets, concentrations of CRP, SAA, and MMP3, and the number of tender joints. On the other hand, H*ε*dC levels showed a significantly positive correlation with the number of swollen joints (*r* = 0.57, *P* < 0.0001) and the total number of swollen and tender joints (*r* = 0.48, *P* = 0.0005) (Table 2 and Figure 4) and were also age-dependent (*r* = 0.43, *P* = 0.003) (Table 2 and Figure 3(b)). Finally, H*ε*dC levels showed no associations with disease duration, class and stage, positivity of autoantibodies such as rheumatoid factor and anticitrullinated protein antibody, or use of NSAID, methotrexate, or prednisolone. 

## 4. Discussion

The study presented here demonstrated that the levels of H*ε*dC, a lipid peroxidation-derived DNA adduct, were significantly higher in the whole blood of RA patients than in that of controls and that this difference increased with aging. To the best of our knowledge, this is the novel finding. In addition, the strong correlation between H*ε*dC levels and the number of swollen joints suggests that H*ε*dC may play a pathological role in the development of RA. 

The etiology of RA has not been fully clarified, but it is generally accepted that interaction between genetic predispositions and environmental factors contributes to its development [1, 2]. Several environmental factors such as smoking, infectious agents, environmental toxins, or nutrients have been found to lead to DNA modification, including the enhanced presence of DNA adducts, which affects gene activation or DNA replication [14]. These factors are also believed to have a significant influence on the development of RA. The assessment of redox status in RA has been extensively studied. In particular, a lot of oxidative stress markers, including DNA damage, have been assessed in different samples (blood, urine, and synovial fluid) of RA patients. In previous studies, the relationship between RA and oxidative stress in genomic DNA has been well documented, and ROS, produced by neutrophils infiltrating into the synovial fluid in RA, have been implicated in the pathogenesis of the disease [5, 15–17]. Oxidative products have been also found to be elevated in the lipids and proteins of RA patients [15]. Heightened DNA damage has been demonstrated through assessments by means of alkaline comet assays [5, 6] or indicated by marked elevation of 8-oxo-dG in urine, synovial fluids, and primary blood lymphocytes of RA patients [2, 15, 18]. In our assay, however, we could not detect overaccumulation of 8-oxo-dG in whole blood cells from RA patients. The reason for this discrepancy is as yet unknown, but we presume that it is due to differences in sample sources or assay methods. Previous studies have analyzed 8-oxo-dG concentrations in urine and synovial fluids by using enzyme-linked immunosorbent assay and 8-oxo-dG levels in peripheral blood lymphocytes by means of high-performance liquid chromatography, while we examined 8-oxo-dG levels in genomic DNA from whole blood cells by means of LC-MS/MS. Another more likely reason is the difference in disease activity, since that of RA patients enrolled in our study appeared to be milder since the median number of tender and swollen joints was 2 and 2, respectively, and the median CRP value was 0.83 mg/dL, values which are lower than those previously reported. However, it should be pointed out again that even in RA patients with mild disease activity, H*ε*dC levels were elevated and correlated well with the number of involved joints, raising a possibility that H*ε*dC may become a novel and sensitive biomarker to detect disease activity of RA.

Analysis of the chemical structure suggests that oxidative DNA damage leads to DNA strand breakages including double- and single-strand breaks and changes in the DNA quaternary structure resulting in its unwinding, and enhanced DNA unwinding has been found in the blood mononuclear cells of patients with RA [19]. The importance of lipid peroxidation-derived DNA damage in inflammatory diseases has been implied [20], and, in fact, levels of lipid peroxidation and oxidised low-density lipoprotein are highly expressed in RA patients, while a positive association between RA disease activity and lipid peroxidation has been reported [10, 15, 21]. Polyunsaturated fatty acids (PUFAs) exerting oxidative stress have been found to cause a series of *α*,*β*-unsaturated aldehydes such as acrolein, crotonaldehyde, and malondialdehyde to form lipid peroxyl radicals, which are highly DNA- and protein-reactive [7, 10, 14, 22, 23]. H*ε*dC has an exocyclic ring and a bulky side chain formed by deoxycytidine reacting with 4-ONE and may lead to alterations in mitochondrial function [7, 14, 22–25]. The presence of H*ε*dC has been identified in various cells and tissues [7]. Functionally, H*ε*dC was found to block DNA synthesis and thus resulted in marked miscoding during the replication of DNA plate modified with deoxycytidine [26]. Williams et al. suggested that either arachidonic acid or linoleic acid is catalyzed by Cox-2 into 15(*S*)-hydroperoxy-5*Z*,8*Z*,11*Z*,13*E*-eicosatetraenoic acid (15(*S*)-HPETE), which then undergoes hemolytic decomposition to form the DNA-reactive bifunctional electrophile, 4-ONE [20]. Considering our findings on the correlation analysis, H*ε*dC may be in part responsible for the pathological activities in synovium, and further studies with the use of synovium are required. It is conceivable that the high-level accumulation of H*ε*dC on RA patients was due to imbalance of oxidative reaction over antioxidant defence system, impairment of DNA repair enzymes and/or excessive activity of 15-lipooxygenase or Cox-2, or the excess presence of 4-ONE or *ω*6 PUFAs in cell membranes. However, no differences were observed in the H*ε*dC levels of RA patients even if they had been treated with NSAID or corticosteroid, which is known to suppress 15-lipoxygenase. RA patients treated with methotrexate reportedly show reduced concentrations of 8-oxo-dG [27], but we could not detect any association between H*ε*dC and methotrexate use. Although it is important to monitor H*ε*dC levels following treatment in order to evaluate the effect of these drugs on H*ε*dC production, our findings also suggest that H*ε*dC is regulated differently from 8-oxo-dG in RA patients. Further studies are required to clarify the mechanism, which accounts for excessive production of H*ε*dC of RA patients.

Although it is clear that H*ε*dC levels in whole blood cells correlate well with the number of involved joints, the pathological effect of increased levels of H*ε*dC in RA remains to be elucidated. Moreover, it is another important issue to clarify whether or not this elevation is specific for RA. Since H*ε*dC may epigenetically alter gene accumulation and is also known to be highly mutagenic [14], this contributes to the persistent inflammation or, through p53 mutations or alternation of gene expression, may be associated with an increase in the incidence of cancer seen in RA patients [28, 29]. Further studies will be required to clarify the role of H*ε*dC in the development of RA and its complications. 

## 5. Conclusion

The present study shows that the lipid peroxidation-derived DNA adduct, H*ε*dC, is highly accumulated in whole blood cells of RA and its level is age dependent. The positive association between H*ε*dC values and the number of involved joints suggests that H*ε*dC may become a novel biomarker to evaluate disease activity of RA. Based on the previous findings and this paper, DNA damages may play a significant role in the development of RA and/or its complications although further studies are required to elucidate the exact significance of DNA adducts in RA. 

## Figures and Tables

**Table 1:** Characteristics of RA patients and controls enrolled in the study.

	RA patients	Controls
Number of subjects	46	31
Females	38 (83%)	14 (45%)
Age (years, median (range))	52 (22–81)	36 (22–57)
Disease duration (years)	10 (1–40)	
Use of NSAIDs	41%	
(Nonselective Cox-2 inhibitor 39%, celecoxib 2%)		
Use of MTX	63%	
Dosage of MTX (mg/week)	6 (0–12)	
Use of prednisolone	63%	
Dosage of prednisolone (mg/day)	3 (0–7.5)	
Use of biologics	11%	
Number of tender joints	2 (0–28)	
Number of swollen joints	2 (0–12)	
Class	2 (1–3)	
Stage	3 (1–4)	
Positive for RF	95%	
Positive for ACPA	93%	
CRP (mg/dL)	0.83 (0.04–6.2)	

NSAIDs: nonsteroidal anti-inflammatory drugs; Cox-2: cyclooxygenase 2; MTX: methotrexate; RF: rheumatoid factor; ACPA: anticitrullinated protein antibody.

**Table 2:** Correlation of H*ε*dC with clinical and laboratory parameters.

Correlation of H*ε*dC with	Number of subjects	Spearman rank correlation (CI)	*P* value
CRP	43	0.19 (−0.11, 0.47)	0.21
PLT	39	0.21 (−0.11, 0.49)	0.20
SAA	15	0.20 (−0.35, 0.64)	0.47
Albumin	15	−0.24 (−0.67, 0.31)	0.37
MMP3	26	0.14 (−0.26, 0.50)	0.50
SJC	46	0.57 (0.34, 0.74)	<0.0001
TJC	46	0.26 (−0.03, 0.51)	0.08
S&TJC	46	0.48 (0.22, 0.68)	0.0005
Age	46	0.43 (0.15, 0.64)	0.003

The Spearman rank correlation coefficient was used for statistical analyses of the associations between H*ε*dC levels and clinical or laboratory test findings for RA patients. *P* < 0.05 was considered significant. CI: confidence interval; PLT: platelet count; SAA: serum amyloid A; MMP3: matrix metalloproteinase 3; SJC: the number of swollen joints; TJC: the number of tender joints; S&TJC: the number of swollen and tender joints.
